# Disseminating a smoking cessation intervention to childhood and young adult cancer survivors: baseline characteristics and study design of the partnership for health-2 study

**DOI:** 10.1186/1471-2407-11-165

**Published:** 2011-05-11

**Authors:** Janet S de Moor, Elaine Puleo, Jennifer S Ford, Mark Greenberg, David C Hodgson, Vida L Tyc, Jamie Ostroff, Lisa R Diller, Andrea Gurmankin Levy, Kim Sprunck-Harrild, Karen M Emmons

**Affiliations:** 1 Division of Health Behavior and Health Promotion, The Ohio State University College of Public Health, Columbus, OH, USA; 2 Department of Public Health, University of Massachusetts, Amherst, MA, USA; 3 Department of Psychiatry and Behavioral Sciences, Memorial Sloan-Kettering Cancer Center, New York, NY, USA; 4 Division of Haematology/Oncology, The Hospital for Sick Children, Toronto, Ontario, Canada; 5 Department of Radiation Oncology, Princess Margaret Hospital and The University of Toronto, Toronto, Ontario, Canada; 6 Department of Psychology, St. Jude Children's Research Hospital, Memphis, TN, USA; 7 Department of Pediatric Oncology, Dana-Farber Cancer Institute, Boston, MA, USA; 8 Department of Medical Oncology and Population Sciences, Harvard School of Public Health and Dana-Farber Cancer Institute, Boston, MA, USA; 9 Department of Medical Oncology and Population Sciences, Dana-Farber Cancer Institute, Boston, MA, USA

## Abstract

**Background:**

Partnership for Health-2 (PFH-2) is a web-based version of Partnership for Health, an evidence-based smoking cessation intervention for childhood cancer survivors. This paper describes the PFH-2 intervention and baseline data collection.

**Methods:**

374 childhood and young adult cancer survivors were recruited from five cancer centers and participated in the baseline assessment. At baseline, participants completed measures of their smoking behavior, self-efficacy and stage of change for quitting smoking as well as psychological and environmental factors that could impact their smoking behavior.

**Results:**

At baseline, 93% of survivors smoked in the past seven days; however, 89% smoked a pack or less during this period. Forty-seven percent were nicotine dependent, and 55% had made at least one quit attempt in the previous year. Twenty-two percent of survivors were in contemplation for quitting smoking; of those 45% were somewhat or very confident that they could quit within six months. Sixty-three percent were in preparation for quitting smoking; however, they had relatively low levels of confidence that they could quit smoking in the next month. In multivariate analyses, stage of change, self-efficacy, social support for smoking cessation, smoking policy at work and home, fear of cancer recurrence, perceived vulnerability, depression, BMI, and contact with the healthcare system were associated with survivors' smoking behavior.

**Discussions/Conclusions:**

A large proportion of the sample was nicotine dependent, yet motivated to quit. Individual- interpersonal- and environmental-level factors were associated with survivors' smoking behavior. Smoking is particularly dangerous for childhood and young adult cancer survivors. This population may benefit from a smoking cessation intervention designed to build self-efficacy and address other known predictors of smoking behavior.

## Background

It is estimated that there are 328,656 survivors of childhood cancer in the United States [1]. Due to advances in cancer treatment, 80% of children diagnosed with cancer will survive longer than five years [2]. The treatments for childhood cancers can precipitate late-onset health problems such as damage to vital organs and treatment-related second cancers; smoking may exacerbate the late effects of cancer treatment [3-9]. The care and management of survivors now includes prevention of adverse late effects and second primary cancers. It is critical that this group refrain from tobacco use and other health-risk behaviors that may further increase their risk for health problems.

Studies designed to describe smoking behavior among childhood cancer survivors have found that this population has lower smoking rates than the general population [10] and age-matched controls [11]. However, a substantial number of childhood cancer survivors still smoke [12-14]. For example, among participants in the Childhood Cancer Survivors Study (CCSS), a collaborative, multi-institutional study comprised of long-term survivors of childhood cancer, 28% reported ever smoking and 17% were current smokers [12]. Data from the population-based cohort in the UK, The British Childhood Cancer Survivor Study, found that approximately 20% were current smokers [15]. These data indicate that a large number of childhood cancer survivors smoke, and interventions are needed to address tobacco use in this high-risk population.

Partnership for Health (PFH), a randomized smoking cessation intervention, was designed to increase survivors' motivation and self-efficacy to quit smoking [12]. Participants were randomized to one of two conditions: (1) self-help (SH) and peer-delivered telephone counseling (PC). Participants in the SH condition received a letter from the study physician about the importance of not smoking and a smoking-cessation manual developed specifically for survivors. Participants in the PC condition received a written feedback report tailored to their unique cancer history and smoking behaviors, up to six peer-delivered telephone counseling calls, and free nicotine replacement therapy for themselves and spouse/significant other if they wanted to quit. PFH effectively decreased tobacco use: the PC condition had a significantly higher quit rate compared with the SH condition at the eight month (16.8% v 8.5%; p < .01) and 12 month (15% v 9%; p < .01) follow ups [16]. In addition, long-term smoking cessation (assessed 2-6 years after baseline) was significantly higher in the PC vs. SH condition [17]. Details of PFH have been published elsewhere [18].

The efficacy of PFH suggested that this program could benefit the broader population of childhood cancer survivors who smoke, thus addressing a need for services not consistently provided by the majority of pediatric cancer treatment facilities [19]. However, research is needed to determine how to disseminate PFH, while maximizing intervention efficacy. PFH-2 was developed to test a web-based version of PFH, which could be disseminated broadly through existing cancer survivorship clinics and other settings, in comparison with the PFH print materials. Web-based interventions have several potential advantages. First, they enable researchers to easily tailor intervention content based on participants' characteristics, which has been shown to improve intervention efficacy [20]. In addition, web-based interventions make it possible to deliver both static information and interactive content to a geographically diverse study population. Further, there is some evidence that web-based smoking cessation interventions, are more effective at changing behavior than other interventions [21]. The goals of this paper are to describe the PFH-2 intervention, present data on the baseline characteristics of the study population, and explore psychosocial and environmental factors associated with childhood cancer survivors' baseline smoking rate, nicotine dependence, and quit attempts.

## Methods

### Eligibility Criteria and Sample Recruitment

PFH-2 was conducted in collaboration with five cancer centers in the United States and Canada: St. Jude Children's Research Hospital, Memorial Sloan Kettering Cancer Center, Princess Margaret Hospital, The Hospital for Sick Children, and Dana-Farber Cancer Institute (DFCI)/Partners. To be eligible, survivors had to be diagnosed with cancer before age 35, currently between ages 18-55, not currently treated for cancer and out of treatment for ≥ two years, mentally able to provide informed consent, reachable by telephone, able to speak English, and a current smoker (defined as having smoked at least one puff in the last 30 days).

This study was approved by the institutional review boards at all participating institutions. Baseline data for PFH-2 were collected from 2005-2008. A preliminary screen for eligiblity was performed at each site. Due to the variability in institutional implementation of patient privacy and Institutional Review Board requirements, the recruitment procedures varied across institutions. However, across all sites, potentially eligible survivors were sent an introductory letter about the study and were given the opportunity to opt-out of further contact by calling a site-specific project phone number mentioned in the introductory letter. After consent was obtained, contact information was forwarded to the study survey team, who verified eligibility and administered the baseline survey. The study was also advertised on Web sites designed for and about childhood and young adult cancer survivors and survivorship (e.g., American Cancer Society, Planet Cancer, The Doug Ulman Fund), and survivors could proactively contact the study team and give verbal consent.

The introductory letter was sent to 4,345 cancer survivors and 4,312 did not opt out of further contact. In addition, 54 people contacted the study directly from postings on the Websites. Of the 4,348 survivors who were contacted, 18% (n = 733) were alive and eligible (ineligibility largely due to smoking status), and 48% (n = 374) of eligible survivors were enrolled in the study.

### PFH-2 Study Design

PFH-2 is a randomized controlled trial with a two-group stage stratified design and the individual as the primary sampling unit. After completing the baseline survey, participants are randomized to one of two intervention conditions, stratified by study site: (1) Web Intervention or (2) Print Materials Intervention.

#### Web Intervention

The Web Intervention includes: (1) a letter encouraging smoking cessation from the site oncologist, developed based on the principles of the NCI's 5 A's smoking counseling guidelines; (2) free pharmacotherapy for participants and their spouses/partners; and (3) a tailored web-based intervention.

Participants randomized to the tailored web-based intervention are assigned a unique username and password to access the site. This will enable us to track their progress through the site including time spent on the website and use of different website features. Although participants always have access to the entire website, they are directed through seven discrete sessions designed to parallel the counseling sessions of the original PFH study.

The web intervention was guided by principles from Social Cognitive Theory, The Transtheoretical Model, and the Precaution Adoption Process Model [22-24]. The goals of the intervention are as follows: (1) assess and enhance participants' motivation to change, (2) address participants' ambivalence about behavior change; (3) provide social support, (4) assess and build participants' self-efficacy; (4) increase participants' awareness of risks; (5) help participants identify and address barriers to change; and (6) address participants' nicotine dependence. To meet these goals, intervention content is tailored based on participants' motivation to quit smoking. Participants can reassess their stage of readiness to quit at any point during the intervention; and at weeks 10 and 18 participants will be prompted to reassess their stage of readiness before entering the website. If participants move to an earlier or later stage of readiness, they will automatically be given the version of the website that is tailored to their current level of motivation to quit smoking.

The site is designed to take participants back to key pieces that they had not yet navigated, depending on their stage of readiness for smoking cessation. However, participants can also override the system and visit all parts of the website. In addition, each time a participant logs into the site, the system gives them feedback showing them where they have been and suggests possible next stops. Fresh content posted to the website and content that the participant has not seen is also highlighted. The website also includes extensive information about how to live a healthy lifestyle, news stories that are relevant to childhood cancer survivors, information about advances in treatment for childhood cancer, late effects, and other relevant medical topics as well as and a forum where study participants have the option of sharing their thoughts and ideas about different topics.

To replicate the peer-to-peer nature of PFH, the Web intervention uses a peer counselor trained in motivational interviewing to moderate the web site's discussion forum and serve as a resource for participants to contact with questions. Scheduled reminder emails and a bi-weekly newsletter about new study findings published in cancer survivorship are used to encourage frequent use of the website.

#### Materials Intervention

The Materials Intervention includes the following: (1) a letter encouraging smoking cessation from the site oncologist, developed based on the principles of the NCI's 5 A's smoking counseling guidelines; (2) free pharmacotherapy for participants and spouses/significant others who want to quit; and (3) tailored and targeted self-help materials addressing participant-specific barriers to change and other survivor-related topics of interest.

### Data Collection Procedures

Baseline data were collected by telephone prior to intervention condition assignment. These data were used to characterize participants' smoking behavior, self-efficacy and stage of change for quitting smoking as well as psychological and environmental factors that could impact their smoking behavior.

## Measures

### Socio-Demographic Characteristics, Body Mass Index (BMI), and Medical History

The following demographic data were collected: age, gender, race, ethnicity, marital status, education, and employment status. We also collected self-reported data on height and weight in order to calculate BMI. In addition, self-reported data were also collected on participants' type of cancer and cancer treatment.

*Internet access and utilization *were measured with a series of questions about whether participants owned a computer, whether they had access to the internet at home and/or work, how frequently they used a computer, and how frequently they checked e-mail or used the internet.

### Smoking behavior

Smoking rate: participants reported the number of cigarettes they smoked per day. Nicotine dependence: participants reported the number of minutes after waking that they smoked their first cigarette [25]; responses were dichotomized as < 30 minutes (nicotine dependent) and ≥ 30 minutes (not nicotine dependent). Quit attempts: participants reported the number of times in the previous 12 months that they tried to quit smoking and stayed off cigarettes for at least 24 hours. Use of pharmacotherapy was assessed with two questions about whether participants had ever used Zyban or nicotine replacement therapy to quit smoking.

### Smoking-Related Motivational Variables

The Stages of Change Scale was used to assess motivation to quit smoking [26], according to four categories: (1) precontemplation: not seriously thinking about quitting smoking in the next six months; (2) contemplation: seriously thinking about quitting smoking in the next six months; (3) preparation: intending to quit smoking in the next month and those who have tried to quit in the past year; (4) action: not currently smoking and quit within the past six months or maintenance: have not smoked for at least six months. Self-efficacy was assessed with two questions about participants' level of confidence that they could quit smoking in six months and one month respectively [18].

### Social Environment

Social support for smoking cessation was assessed with a series of questions about whether participants' family, friends, coworkers, and health care providers encouraged them to quit smoking. We also asked whether participants' spouse or partner smoked [16]. Household and workplace smoking policy: Participants were asked to characterize the rules about smoking at home and work. Response options captured whether smoking was unrestricted inside the building/house, limited to certain rooms, or forbidden inside the building/house.

### Psychological Variables

Fear of cancer recurrence was assessed with a question about whether in the previous week, participants worried about getting cancer again. Fear of recurrence was also assessed with the Intrusive Thoughts sub-scale of the Impact of Events Scale (IES), which measures the frequency with which thoughts intrude into consciousness [27]. Perceived control was assessed with the 3-item Perceived Control Scale, which measure the degree to which participants felt they could control the physical side effects, future health, and chance of a cancer recurrence [28]. Perceived vulnerability was assessed with a question about perceived risk of any serious future health problem. Perceived vulnerability was also assessed with the Perceived Importance of Health Protection scale, which assesses survivors' perceptions of their vulnerability to tobacco-related health risks relative to age and gender matched peers [29]. Depression was measured with the 2-item Prime MD scale, which asks about feelings of depression and loss of interest or pleasure during the previous month [30]. Respondents who answered "yes" to either question were classified as screening positive for possible depression [30].

### Contact with the Healthcare System

Participants were asked whether they had a regular healthcare provider and whether they had been seen by their primary care physician or their oncologist in the past year. Participants were also asked if they had been hospitalized in the past year.

### Data Analysis

Descriptive analyses were used to characterize the study population. Multinomial logistic regression was used to identify predictors of participants' smoking rate, nicotine dependence, and number of quit attempts in the past year. A step-wise selection approach was used to enter variables into the model. Variables significant at p ≤ .25 in the bivariate analysis were entered into the multivariate model. Those variables that remained significant at p ≤ .15 were retained in the model. The following predictors, selected based on our and others' previous work, were examined: BMI, stage of change, self efficacy, social support for smoking cessation, fear of cancer recurrence, intrusive thoughts, perceived control, perceived vulnerability, depression, contact with the healthcare system and smoking policy at work and home. Smoking behavior differed by recruitment site, cancer treatment, and time since diagnosis, so we adjusted for these variables in our analysis. In addition, we adjusted for age, gender, race and education, cancer diagnosis, as well as the two smoking behaviors not being analyzed.

## Results

### Participant Characteristics

The mean age at enrollment was 32.4 (σ = 7.94). The sample was 51% male, 86% white, and 30% had at least a college degree. In addition, 48% were married or living with a partner, and 80% were employed in the last year. The mean years since cancer diagnosis was 20 (σ = 9.61), and leukemia was the most common type of cancer diagnosis reported by study participants (23%). Eighty-one percent of the sample owned their own computer, 82% had access to the Internet at home and/or work, and 77% used the Internet at least once per week.

### Smoking Behavior and Stage of Change

Descriptive statistics for smoking behavior, stage of change, and self-efficacy are presented in Table 1. Over 92% of participants smoked in the last seven days and 89% smoked less than one pack of cigarettes during this time period. The median number of cigarettes smoked per day was 15. Forty-seven percent of the sample was nicotine dependent, and the majority was thinking about quitting smoking--only 15% were in pre-contemplation, 22% were in contemplation and 63% were in preparation. Among those in contemplation, 45% were somewhat or very confident that they could quit smoking in the next six months. Among those in preparation, 40% were somewhat or very confident that they could quit smoking in the next month. Because of our eligibility criteria, no participants were in the stages of action or maintenance at baseline.

**Table 1:** Frequency of smoking behavior, stage of change, and self-efficacy for smoking cessation (N = 374)

	N	%
Smoked in the past 7 days	346	92.51
Average number of cigarettes smoked in past 7 days^1^		
0-4	84	22.46
5-9	57	15.24
10-14	67	17.91
15-20	100	26.74
>20	39	10.43
Number of minutes after waking until first cigarette^2^		
< 30	126	33.69
≥ 30	242	64.71
Number of quit attempts in previous 12 months^3^		
0	162	44.88
1-3	131	36.29
≥4	68	18.84
Tried NRT^4 ^to quit smoking	133	35.56
Tried Zyban to quit smoking	41	10.99
Contemplation stage of change (e.g., seriously thinking about quitting smoking in the next 6 months)	312	84.78
Preparation stage of change (e.g., seriously thinking about quitting smoking in the next month)	230	62.50
Self-efficacy to quit in the next 6 months		
Not at all confident	33	8.87
A little confident	54	14.52
Somewhat confident	117	31.45
Very confident	83	22.31
Extremely confident	85	22.85
Self-efficacy to quit in the next month		
Not at all confident	84	22.58
A little confident	82	22.04
Somewhat confident	87	23.39
Very confident	60	16.13
Extremely confident	59	15.86

^1 ^6 missing

^2 ^4 missing

^3 ^13 missing

^4 ^NRT = nicotine replacement therapy

### Multivariate Analysis

#### Smoking rate

In the model to predict the number of cigarettes smoked per week, a more lenient smoking policy at work, encouragement to quit smoking from one's family and oncologist and relatively low self-efficacy to quit smoking were all associated with increased odds of smoking more cigarettes, whereas being hospitalized for a medical problem was associated with a decreased odds of smoking more cigarettes (Table 2). Stage of change, household smoking policy, fear of cancer recurrence, intrusive thoughts, perceived control, perceived vulnerability, BMI and contact with the healthcare system were not associated with smoking rate.

**Table 2:** Odds ratios predicting number of cigarettes smoked per week (n = 336)^1, 2^

	> 10 v. ≤ 5	>10 v. 6-10	6-10 v. ≤ 5
	OR (95% CI)	OR (95% CI)	OR (95% CI)
Hospitalized for medical problem (no = ref)	0.37 (0.10, 1.37)	0.19 (0.06, 0.60)	1.92 (0.61, 6.04)
Depression (no = ref)	2.39 (0.98, 5.83)	0.90 (0.42, 1.91)	2.66 (1.15, 6.16)
Smoking policy at work (forbidden to smoke inside = ref)			
There are no rules	2.84 (0.71, 11.40)	1.91 (0.55, 6.61)	1.49 (0.44, 5.01)
People can only smoke in certain rooms or areas	7.65 (1.74, 33.64)	1.43 (0.50, 4.08)	5.34 (1.13, 25.19)
Did not work in past year	0.29 (0.09, 0.91)	1.18 (0.46, 3.05)	0.24 (0.08, 0.73)
Social support from family (ref = no).	6.48 (2.24, 18.80)	1.41 (0.55, 3.62)	4.58 (1.62, 12.98)
Social support from oncologist (ref = no)	3.40 (1.24, 9.34)	0.58 (0.26, 1.32)	5.83 (2.17, 15.65)
Self-efficacy: Level of confidence to quit smoking in 6 months (ref = very or extremely confident)			
Not at all confident	5.28 (0.54, 51.68)	1.55 (0.25, 9.23)	3.41 (0.35, 33.22)
A little or somewhat confident	1.26 (0.44, 3.61)	0.33 (0.12, 0.89)	3.85 (1.34, 11.16)
Self-efficacy: Level confidence to quit smoking in 1 month (ref = very or extremely confident)			
Not at all confident	4.60 (0.99, 21.42)	1.44 (0.32, 6.48)	3.20 (0.77, 13.30)
A little or somewhat confident	8.78 (2.39, 32.25)	2.71 (0.83, 8.92)	3.23 (1.00, 10.46)

^1^Adjusted for recruitment site, age, gender, race, education, treatment, diagnosis, nicotine dependence and quit attempts.

^2 ^n = 102 survivors smoked ≤ 5 cigarettes per day; n = 90 survivors smoked between 6-10 cigarettes per day; and n = 155 survivors smoked >10 cigarettes per day.

#### Nicotine dependence

In the model to predict nicotine dependence, lack of healthcare provider for non-emergency care, being hospitalized in the past year, no fear of cancer recurrence, no rules about smoking in the house, and low self-efficacy to quit smoking in the next month were all associated with increased odds of being nicotine dependent (Table 3). Stage of change, social support for smoking cessation, workplace smoking policy, intrusive thoughts, perceived control, perceived vulnerability, depression, and BMI were not associated with the odds of being nicotine dependent.

**Table 3:** Odds ratios predicting nicotine dependence (n = 348)^1^

	Nicotine Dependence
	OR (95% CI)
Health care provider for non-emergency care (yes = ref)	1.96 (0.94, 4.08)
Hospitalized for medical problem in past year (no = ref)	2.56 (1.16, 5.63)
Fear of cancer recurrence (yes = ref)	2.08 (0.81, 5.32)
Smoking policy at home (forbidden to smoke inside = ref)	
There are no rules	2.19 (1.12, 4.26)
People can only smoke in certain rooms or areas	1.94 (0.87, 4.31)
Self-efficacy: level of confidence to quit smoking in 1 month (ref = very or extremely confident)	
Not at all	1.99 (0.84, 4.70)
A little/somewhat	0.74 (0.33, 1.67)

^1^Adjusted for recruitment site, age, gender, race, education, treatment, diagnosis, number of cigarettes smoked per day and quit attempts.

#### Quit attempts

In the model to predict number of quit attempts in the past 12 months, low perceived vulnerability, BMI, social support for smoking cessation from participants' regular doctor and oncologist and being in a later stage of change were associated with an increased odds of making more quit attempts (Table 4). Self-efficacy, workplace and household smoking policy, fear of cancer recurrence, intrusive thoughts, perceived control, depression, and contact with the healthcare system were not associated with number of quit attempts.

**Table 4:** Odds ratios predicting quit attempts during the past 12 months (n = 339)^1^

	Quit Attempts During the Past 12 Months		
	>4 vs 0	> 4 vs 1-3	1-3 vs 0
	OR (95% CI)	OR (95% CI)	OR (95% CI)
Perceived vulnerability (≤ unlikely = ref)			
Moderate chance	0.98 (0.34, 2.77)	0.95 (0.36, 2.49)	1.03 (0.46, 2.30)
Likely	0.47 (0.15, 1.46)	0.49 (0.16, 1.48)	0.96 (0.42, 2.19)
Very likely/certain to happen	0.73 (0.26, 2.02)	2.05 (0.71, 5.95)	0.35 (0.15, 0.85)
BMI	1.09 (1.02, 1.16)	1.05 (0.98, 1.12)	1.04 (0.99, 1.09)
Social support from regular doctor (no = ref)	1.28 (0.59, 2.79)	0.57 (0.27,1.21)	2.23 (1.22, 4.10)
Social support from oncologist (no = ref)	3.08 (1.30, 7.27)	2.71 (1.12, 6.57)	1.14 (0.56, 2.29)
Stages of change (precontemplation = ref)			
contemplation	1.47 (0.29, 7.47)	0.41 (0.07, 2.47)	3.57 (1.26, 10.06)
preparation	11.34 (2.88, 44.67)	1.69 (0.38, 7.46)	6.70 (2.56, 17.54)

^1^Adjusted for recruitment site, age, gender, race, education, treatment, diagnosis, number of cigarettes smoked per day and nicotine dependence.

## Discussion/Conclusions

Over 90% of our sample of childhood and young adult cancer survivors smoked within the past seven days, but overall the smoking rate of the sample was light, with the vast majority smoking less than one pack per week. However, almost half of the participants were classified as nicotine dependent. Participants in the current study smoked fewer cigarettes than has been found in other studies of childhood cancer survivors [15,18]. Although we can not definitely determine the reason for this difference, there are several possible explanations. First, several years have passed since the last report of childhood cancer survivors' smoking behavior was published. National data suggests that the prevalence of tobacco use has decreased during this period for the United States population and for cancer survivors as a collective group [31,32]. Smoking rates for childhood and young adult cancer survivors could have declined at an accelerated rate, which would be consistent with historical data on tobacco use trends among young adults [32]. Second, data on the late effects of cancer treatment are increasingly becoming available. Childhood and young adult cancer survivors may be responding to these data by limiting their tobacco use to decrease their risk for future health problems. Further, survivors who are nicotine dependent may be more likely to reduce their smoking rate, rather than quit smoking, to minimize their risk for late effects, which explains why we saw high rates of nicotine dependence (65%) despite a low smoking rate. Third, the low smoking rate may reflect survivors' efforts to quit as data suggest that some smokers will decrease the amount of cigarettes they smoke before quitting completely[33]. Finally, the low smoking rate may also reflect selection bias such that lighter smokers may have been more willing to participate in a smoking cessation intervention. Regardless, there is no research on suggesting that even light smoking is safe for childhood cancer survivors; thus, it is important to help this high-risk population refrain from tobacco use completely.

The majority of our sample was motivated to quit smoking--22% were in contemplation and 63% were in preparation. However, less than one half of participants in contemplation and preparation were very or extremely confident that they could quit. Over half of survivors made at least one quit attempt in the previous 12 months and about 20% made four or more quit attempts. Collectively, these data suggest that childhood and young adult cancer survivors in this study are motivated to quit, and may benefit from a targeted and tailored smoking cessation intervention to help them build self-efficacy and quit successfully. We anticipate that the web-based format of PFH-2 will be an accessible delivery channel for this population because of the widespread access and utilization of the Internet.

In our multivariate analysis of the baseline data, stage of change and self-efficacy were associated with smoking behaviors. Cancer survivors in an early stage of change for smoking cessation made fewer attempts to quit smoking. Further, survivors with low self-efficacy to quit smoking smoked more cigarettes per week, and there was evidence that they were more likely to be nicotine dependent. These results are consistent with research finding that a later stage of change and high self-efficacy were consistently associated with smoking cessation [34] as well as literature on the importance of tailoring smoking cessation interventions to smokers' stage of change [35].

Aspects of the social environment were also associated with smoking behavior. Survivors who smoked more were more likely to have received social support to quit smoking from their family and oncologist. It is possible that survivors who smoked more may have triggered family and health care providers to encourage them to quit smoking. In fact, encouragement to quit smoking by one's regular doctor or oncologist was associated with a making more quit attempts. These data are consistent with Emmons et al., (2003) who found that social support for quitting smoking was associated with higher smoking rates but more quit attempts [18].

Other evidence for the importance of the social environment came from our data on household and workplace smoking policies. Survivors with a less stringent smoking policy at work were more likely to smoke more, whereas survivors with a less stringent smoking policy at home were more likely to be nicotine dependent. Workplace smoking bans make it more difficult to smoke during the day and may facilitate social norms around not smoking. Moreover, people employed in workplaces with smoking bans are more likely to smoke fewer cigarettes and think about quitting [36]. Household smoking bans may reflect pressure from the smoker's family for them to quit or the smoker's readiness to quit. Regardless, smokers who enact household smoking bans are more likely to make successful quit attempts and have lower risk of relapse [37,38].

Survivors' psychosocial characteristics were also associated with their smoking behavior. Survivors who did not fear cancer recurrence were more likely to be nicotine dependent; and survivors who felt more certain that they would experience a serious future health event made fewer quit attempts. These findings appear somewhat contradictory. The literature suggests that survivors with lower risk perception may be less motivated to change their smoking behavior than smokers with a greater perceived risk of future health problems [39], which is consistent with our finding for cancer recurrence. However, it is possible that survivors who feel relatively certain that they will experience a future health event are demonstrating fatalistic beliefs about their future health potential, which are associated with a increased likelihood of smoking and an earlier stage of change for smoking cessation [40].

Survivors who had been hospitalized for a medical problem in the last year were less likely to be relatively heavy smokers but more likely to be nicotine dependent. In addition, survivors who lacked a healthcare provider for non-emergency care were more likely to be nicotine dependent. Survivors who do not have access to a primary care provider (PCP) may not receive consistent messages about the importance of making healthy lifestyle choices such as quitting smoking. In addition, survivors who lack a PCP may not have the same level of access to pharmacotherapy and behavioral interventions for smoking cessation as survivors with a PCP.

Our findings provide important information about factors that are associated with smoking behavior among childhood cancer survivors. In addition, our results indicate that smoking cessation interventions developed for childhood cancer survivors need to enhance motivation to quit by providing accurate information about the relationship between smoking and future health problems as well as the fact that smoking is particularly harmful for cancer survivors. Coupled with efforts to increase survivors' perceived susceptibility to tobacco-related health problems, interventions must help survivors build self-efficacy for quitting smoking by addressing their psychological barriers to quitting, increasing social support for smoking cessation, and targeting aspects of the social environment that can constrain smoking behavior (e.g., household smoking policy). In addition, even though study participants tended to be light smokers, there are high rates of nicotine dependence in this group, suggesting that pharmacotherapy may facilitate quitting.

### Limitations

This study had several limitations. First, we were unable to contact 24% of survivors who were identified by participating sites and did not opt out, and we do not know whether these individuals were eligible for the study. Our study population is young adults who tend to be very mobile and likely living in a different location from the time of their initial treatment. In many cases, the participating sites did not have accurate addresses on file for their patients. We made a concerted effort to locate these individuals by using internet searches and other telephone and mail-based tracing approaches. However, despite this effort, in some cases, our strategies were unsuccessful. Second, 48% of eligible survivors declined to participate, which introduces selection bias into the study; however, a 52% recruitment rate among a sample of smokers who have not sought out smoking cessation treatment is reasonable. Third, our sample may not be representative of all childhood and young adult cancer survivors who smoke because lighter smokers may have been more willing to participate in this study than heavier smokers. Results should be interpreted accordingly. Third, our sample was predominately white, which limits our ability to generalize to other racial and ethnic groups, although it reflects the racial/ethnic characteristics of the participating treatment sites.

### Strengths

This study also had several important strengths. First, study participants were recruited from five pediatric and young adult cancer treatment facilities in the United States and Canada. Thus, our results have higher external validity than if recruited from a single site. Second, we collected data on participants' level of motivation to quit smoking and confidence in their ability to quit smoking in order to tailor intervention content. This will help us evaluate whether the intervention affected theoretical mediators of smoking cessation and better understand the pathways through which the PFH -2 content affected smoking behavior.

## Conclusions

The results of this study suggest that a high proportion of childhood cancer survivors are nicotine dependent, yet motivated to quit. Thus, they may benefit from a targeted and tailored smoking cessation intervention to help them quit successfully. We anticipate that the web-based format of PFH -2 will be an accessible delivery channel for this population because of the widespread access and utilization of the Internet.

## Competing interests

The authors declare that they have no competing interests.

## Authors' contributions

JD participated in the development of this study and drafted the manuscript. EP directed the screening and recruitment of study participants and oversaw the data analysis. She also commented on drafts of the manuscript. JF co-directed the identification of study participants at one of the participating sites and commented on drafts of the manuscript. MG directed the identification of study participants at one of the participating sites and commented on drafts of the manuscript. DH directed the identification of study participants at one of the participating sites and commented on drafts of the manuscript. VT directed the identification of study participants at one of the participating sites and commented on drafts of the manuscript. JO co-directed the identification of study participants at one of the participating sites and commented on drafts of the manuscript. LD directed the identification of study participants at one of the participating sites and commented on drafts of the manuscript. AGL participated in the development of the study and commented on drafts of the manuscript. KSH participated in the development of the study and coordinated study activities. She also commented on drafts of the manuscript. KE was the principal investigator of this study. She directed all study activities and commented on drafts of this manuscript.

All authors read and approved the final manuscript.

## Pre-publication history

The pre-publication history for this paper can be accessed here:

http://www.biomedcentral.com/1471-2407/11/165/prepub

## Ackowledgments

This project was funded by the National Cancer Institute, 5R01CA106914-05.
